# Celiac disease risk varies between birth cohorts, generating hypotheses about causality: evidence from 36 years of population-based follow-up

**DOI:** 10.1186/1471-230X-14-59

**Published:** 2014-04-02

**Authors:** Fredinah Namatovu, Olof Sandström, Cecilia Olsson, Marie Lindkvist, Anneli Ivarsson

**Affiliations:** 1 Department of Public Health and Clinical Medicine, Epidemiology and Global Health, Umeå University, Umeå SE-901 87, Sweden; 2 Department of Clinical Sciences, Paediatrics, Umeå University, Umeå, Sweden; 3 Department of Food and Nutrition, Umeå University, Umeå, Sweden

**Keywords:** Celiac disease, Children, Incidence, Epidemiology

## Abstract

**Background:**

Celiac disease (CD) is a major public health problem with estimated 1-3% prevalence in the general population. In recent years an increase in CD prevalence has been reported both in Sweden and worldwide. This study aimed at examining the annual incidence rate of biopsy-proven celiac disease among children in Sweden over a 36-year period, to assess variations by age, sex and birth cohort, and to assess the clinical impact of these changes.

**Methods:**

The National Swedish Childhood CD Register was used to identify 9107 children aged 0–14.9 years who were diagnosed with CD during the period 1973 to 2009. From 1973 to 1990 the register covered 15% of the nation, this increased to 40% during 1991–1997; a full national coverage was obtained from 1998 onwards. Estimations for the annual incidence rate, cumulative incidence and clinical impact by age groups, calendar month and birth cohorts were made.

**Results:**

CD incidence is continuing to increase in the child population aged 2–14.9 years. A continued variation in CD incidence was observed in children aged 0–1.9 years, characterized by a marked decrease in most recent years. The median age at diagnosis has increased from 1.0 year in the 1970s to 6.8 years in 2009. The average number of new cases has risen from ~200 during 1973–1983 to ~600 during 2004–2009. In the birth cohorts of 2000–2002 the cumulative incidence even exceeded that of the epidemic cohorts at comparable ages. The highest cumulative incidence was observed in the birth cohorts of 1985–1995 and 2000–2002.

**Conclusions:**

CD risk varies between birth cohorts, suggesting cyclic environmental and/or lifestyle risk factors in CD etiology. More research on underlying risk factors is required in order to move forward with preventive strategies.

## Background

Celiac disease (CD) is a chronic small intestinal immune-mediated enteropathy triggered by the ingestion of gluten in genetically susceptible individuals [1]. Children with undiagnosed CD are at risk for stunted growth, delayed puberty, and many other health problems [2,3]. CD is most effectively treated by life-long elimination of wheat, rye, and barley, which results in restoration of the intestinal morphology and alleviation of CD symptoms and some associated health risks [4,5].

The National Swedish Childhood CD Register has tracked the incidence of CD in children below 15 years of age since 1973, revealing a CD epidemic between 1984 and 1994 among children younger than 2 years of age [6,7]. The epidemic was explained by changes over time in infant feeding, and by the interaction between infant feeding and infections early in life [6-9]. The current study aims to examine the annual incidence rate of biopsy-confirmed CD among children in Sweden over a 36-year period and to assess variations by age, sex, and birth cohort as well as the clinical impact of these changes.

## Methods

The National Swedish Childhood CD Register was used to identify children from 0 to 14.9 years of age with newly diagnosed CD. The register was established in 1991 and is hosted by the unit of Epidemiology and Global Health at Umeå University, Sweden, and it currently records all new probable CD cases identified in Sweden. Children diagnosed with CD between 1973 and 1990 were retrospectively added to the register from the records of five pediatric units, covering about 15% of the national population. From 1991 to 1997, 14 pediatric units (40% national coverage) reported their cases to the register. Nationwide coverage was attained in 1998, and all 47 pediatric clinics in Sweden now report cases to the register. Other details regarding the National Swedish Childhood CD Register can be found elsewhere [6].

### Celiac disease case ascertainment

CD was ascertained using the diagnostic criteria provided by the European Society for Pediatric Gastroenterology, Hepatology and Nutrition (ESPGHAN). Before 1990, a CD diagnosis required three consecutive small intestinal biopsies, the first with a normal gluten-containing diet, the second without gluten, and the third after re-introduction of gluten [10]. Revised diagnostic criteria, which were launched in 1990 and fully adopted in Sweden about 10 years later, consider villous atrophy on a normal diet, followed by clinical remission on a gluten-free diet, as sufficient for diagnosis [11].

**Figure 1:**
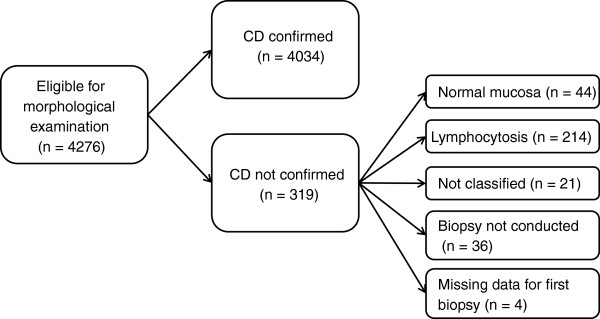
Inclusion and exclusion procedure for CD cases diagnosed from 2004 to 2009.

Using these criteria a total of 9107 children with verified CD were identified during the period from 1973 to 2009. Of these, 2151 were diagnosed from 1973 to 1997, and 2922 cases were identified from 1998 to 2003 [6,12]. During the last follow-up from 2004 to 2009, 4279 children were reported to the register as suspected CD cases. A small intestinal biopsy showing villous atrophy confirmed CD in 4034 of these children, and the remaining 319 were excluded (Figure 1).

### Population data

Population data were obtained from Statistics Sweden and were divided according to hospital catchment area, time period, and age group. The baseline period from 1973 to 1997 consisted of 598,262 births and 8,909,501 person-years of follow-up, the period from 1998 to 2003 had 454,923 births and 9,777,930 person-years of follow-up [6,12] and the period from 2004 to 2009 had 524,909 births and 9,352,310 person-years of follow-up.

### Statistical analysis

The occurrence of CD in children is reported as the incidence rate, cumulative incidence, and clinical impact. Annual incidence rates were calculated by dividing the number of diagnosed new cases by the number of person-years of follow-up, approximated by the mid-year population, and were reported per 100,000 person-years. Age-specific incidence rates were calculated for the age categories 0–1.9, 2–4.9, and 5–14.9 years for the period from 1973 to 2009 to enable comparison with previous studies. Additional incidence rates were calculated for the period from 1998 to 2009, with stratification by age (0, 1, 2, 3–6, 7–10, and 11–14 years), to examine recent changes in detail. Cumulative incidence by age from 1973 to 2009 was calculated by dividing the number of cases diagnosed up to the highest age attained by the number of newborns in each birth cohort, and it was reported per 1000 births. Clinical impact was defined as the annual number of new CD cases diagnosed in the entire country, and was reported for the age categories 0–1.9, 2–4.9 and 5–14.9 years for the period from 1973 to 2009. This was estimated by multiplying each annual age-specific incidence rate by 100,000 person-years, which reflected the average Swedish birth cohort of 100,000 children. The statistical significance of differences in CD incidence rates was determined with a logistic regression model using CD as a binary dependent variable and the year of diagnosis as an explanatory covariate. The log-rank test was used to compare statistical significance in the curves of the cumulative incidence for different birth cohorts. Microsoft Excel 2008 (version 12.3.2) and SPSS 19.0 (SPSS, Chicago, IL) were used for all estimations.

### Ethical considerations

The Research Ethics Committee of all Swedish Medical Faculties approved the use of the National Swedish Childhood CD Register.

## Results

### Annual incidence rates from 1973 to 2009

**Figure 2:**
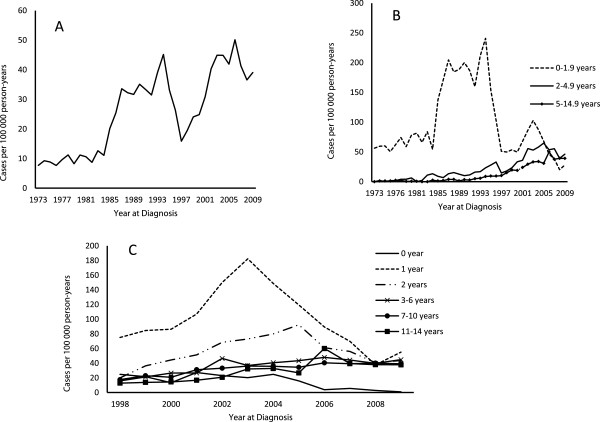
**The annual incidence rate of CD in Swedish children from 1973 to 2009. A)** The average incidence rate for children of all ages. **B)** The incidence rate stratified in three age groups. **C)** More detailed information on the incidence rate for the period from 1998 to 2009.

Over the 36 years of follow-up, there has been an average annual increase in CD incidence in the child population of 4% (Odds ratio = 1.04, *p* <0.001). The average baseline incidence rate in children aged 0–14.9 years was ~10 cases per 100,000 person-years from 1973 to 1984, and this increased to ~33 cases per 100,000 person-years from 1985 to 1994. A temporary decline occurred from 1995 to 1997, with an average incidence rate of 25 cases per 100,000 person-years, but the rate increased again from 1998 to 2003 and from 2004 to 2009, reaching 31 cases per 100,000 person-years and 42 cases per 100,000 person-years, respectively (Figure 2A).

Figure 2B shows a remarkable variation over time in the CD rate in children younger than 2 years of age. In the 1970s, the incidence rate was about 65 cases per 100,000 person-years. The incidence rate increased sharply in the mid 1980s, and during the period from 1985 to 1994 the average incidence rate was 189 cases per 100,000 person-years. This was followed by an abrupt decline starting in 1995, and by 2000 the average incidence rate was about 77 cases per 100,000 person-years. A temporary increase occurred from 2001 to 2003, reaching 85 cases per 100,000 person-years, but the rate declined again during the period from 2004 to 2009 to 48 cases per 100,000 person-years, which was the lowest rate in this age group since surveillance was initiated.

Children aged 2–4.9 years experienced a gradual but persistent increase over time in the incidence rate of CD. We estimated an average incidence rate of 53 cases per 100,000 person-years during the period from 2004 to 2009, a rate ten times higher than that observed during the period from 1973 to 1984 (Figure 2B).

A similar pattern was seen in children aged 5–14.9 years, but it was always at a lower level compared to the younger age groups. Over the period from 1973 to 1984 the average incidence rate was about 1 per 100,000 person-years, and this increased to ~ 4 cases per 100,000 person-years from 1985 to 1994, and continued to rise to 23 cases per 100,000 person-years from 1998 to 2003 (Figure 2B). During the period 2004 to 2009, the mean average incidence rate reached 38 cases per 100,000 person-years.

### Age-specific annual incidence rates from 1998 to 2009

The CD incidence rate varied considerably over the period from 1998 to 2009 for all age groups (Figure 2C). The already low CD incidence rate among children below one year of age decreased further to become negligible. The highest incidence rate was always found in the age group 1–1.9 years and was characterized by an upward trend from 1998 to 2003, followed by an explicit decline from 2004 to 2008 and a slight increase again in 2009. In children 2–2.9 years of age, the incidence rate increased from 1998 to 2005 and then declined, and by 2008 it had reached a level similar to that observed in older children. Throughout this period from 1998 to 2008, the CD incidence rates in children aged 3–6.9, 7–10.9, and 11–14.9 years were about the same and were characterized by a slow but persistent increase over time.

### Clinical impact

**Figure 3:**
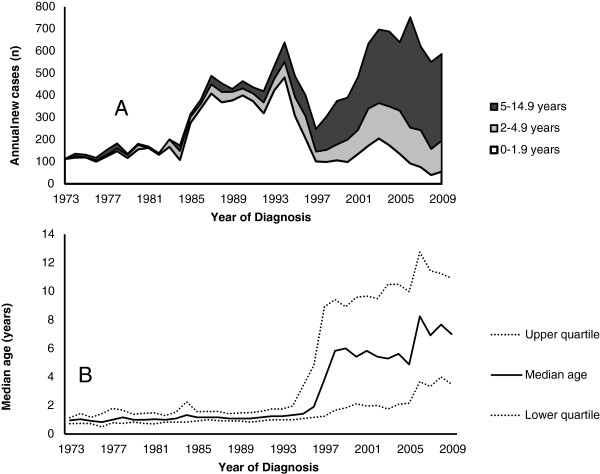
**New cases and age at diagnosis. A)** The estimated number of new CD cases annually in the Swedish childhood population during the period from 1973 to 2009. **B)** The median age at CD diagnosis for children 0–14.9 years of age from 1973 to 2009.

Considering the whole period from 1973 to 2009, the annual number of cases diagnosed increased over time except for a temporary drop during the period 1995 to 1997 (Figure 3A). Between 1973 and 1984, ~ 200 new cases were detected annually and almost all cases were found in children younger than 2 years of age. In the mid-1980s there was an abrupt increase, and the annual number of diagnosed cases was ~500. This rate remained high until the mid 1990s. During this period nearly all new CD cases were still diagnosed in children less than two years of age, but CD had also become slightly more common in those aged 2–14.9 years (Figure 3A). From 1998 to 2009 the annual number of newly detected cases gradually increased up to ~600 cases, and during this time period the number of new cases was highest in children aged 5–14.9 years.

### Age at diagnosis

From 1973 to 1994 the median age at diagnosis, was estimated at 1.1 years, interquartile range 0.8–1.5 years, and it remained very low. Starting in 1995 the median age at diagnosis abruptly shifted to 4.6 years (interquartile range 1.6–8.3 years). The median age at diagnosis has slowly continued to increase up to 6.7 years (interquartile range 3.1–11.1 years) during the period 2004 to 2009.

### Sex-distribution

The incidence rate of CD over the entire follow-up period is about twice as high in girls compared to boys. Among the newly diagnosed children (2004–2009), CD was confirmed in 2546 girls (63%) and 1488 boys (37%). These figures are in accordance with the sex distribution seen in our previous studies covering the period from 1973 to 2003 [6,12].

### Cumulative incidence

**Figure 4:**
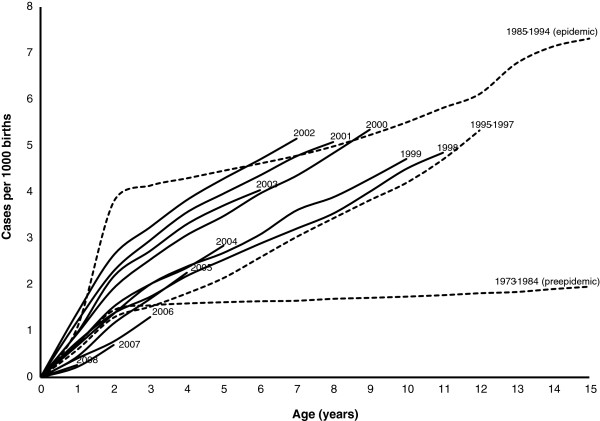
**Cumulative incidence of CD for children born between 1973 and 2009 and followed from birth until 2009 or to an age of 14.9 years.** To increase readability, cohorts from 1973–1997 are aggregated into three intuitive groups according to similarities in the cumulative incidence in those periods. Each cohort from 1998 to 2008 is reported separately.

The epidemic birth cohorts (1984–1994) maintained the highest cumulative incidence at comparable ages over the entire follow-up period until 2009, and the only exceptions to this pattern were the cohorts of 2000–2002 (Figure 4). The epidemic cohorts were characterized by a rapid increase in the cumulative incidence during the first 2 years of age, followed by a gradual but continuous increase up to 14 years of age, which was the highest age for follow-up in this study. None of the post-epidemic birth cohorts have had such a sharp increase in cumulative incidence before 2 years of age, but have instead shown a gradual increase as birth cohorts grow older (Figure 4).

The cumulative incidence of the post-epidemic birth cohorts of 1995–1999 was significantly lower compared to both the epidemic cohorts (1985–1994, *p* <0.001) and the post-epidemic high-incidence cohorts of 2000–2002 (*p* <0.001). As the age of the birth cohorts increased, the gap between the incidence of the epidemic birth cohorts and that of the cohorts of 1995–1999 narrowed. The cumulative incidence of the birth cohorts of 2000–2002 reached, and even exceeded, that of the epidemic cohorts (1985–1994) during the last years of follow-up when these cohorts were 6 to 9 years of age (Figure 4).

The birth cohorts of 2006–2008 diverged from other post-epidemic birth cohorts by having the lowest cumulative incidence up to 3 years of age ever reported since the surveillance program started in 1973. The pre-epidemic cohorts of 1973–1984 still had the lowest reported cumulative incidence over the age span of 4 to 14 years of age.

## Discussion

This study shows considerable changes in CD risk over time in Sweden that are dependent on age and year of birth. Over the 36 years of follow-up we have observed an overall increase in CD risk in the entire child population except for a temporary decline from 1995 to 1997. At the same time, a substantial drop in CD incidence has occurred in the subgroup of children below 2 years of age, with almost zero CD cases diagnosed within the first year of life. The changing CD pattern is also illustrated by a continued increase in the median age at diagnosis. Our findings from cumulative incidence analysis suggest that year of birth may determine specific CD risks, exemplified by excess risk in the birth cohorts of 1985–1995 and 2000–2002. The observed fluctuations in CD cannot be explained by genetic changes, and therefore strongly suggest the role of cyclic environmental and lifestyle risk factors in CD etiology.

The main strength of this study is the identification of CD cases through the Swedish National Childhood CD Register, with a prospective reporting since 1998 from all pediatric departments in the country. Differences in population coverage may not have introduced any major biases, since our findings for the period from 1973 to 1990, with 15% coverage of the total child population are comparable to findings from an earlier national wide study conducted during the same period [13]. We have also previously shown that 40% coverage was sufficient for estimating the national CD incidence [12].

In this study CD diagnosis was based on villous atrophy in order to avoid overestimation of the CD risk. We excluded all cases without villous atrophy even if they had elevated serological markers, minor enteropathy and symptoms typical for CD. The observed overall increase cannot by due to mass screening for the general population since this has not been implemented in Sweden. Additionally, serological testing in genetically susceptible high-risk populations is encouraged and conducted liberally. However, this cannot be solely responsible for the observed increase. The latest ESPGHAN guidelines where CD diagnosis may be based on serological markers were introduced in 2012 [14] and therefore did not influenced our findings since our follow-up ended in 2009.

The limitation of this study is that the CD register primarily captures cases detected clinically. However, it is well established that most CD cases both worldwide and in Sweden remain undiagnosed, and thus most CD studies and knowledge of CD are limited to clinical cases [15,16]. In fact, our recent CD screening study revealed two thirds of all cases were only identified through screening [17].

We found an overall increase in CD incidence in children mainly due to a rising incidence in children aged 2–14.9 years. An increase in CD occurrence [18-23] along with other immune-mediated disorders such as diabetes, asthma, and inflammatory bowel disease, is not unique to Sweden and has been reported elsewhere [24-26]. Increased awareness may be one of the contributing factors to the rising incidence observed in children aged 2–14.9 years. However, this is insufficient in explaining variations seen in the youngest children in whom there has been a sharp increase from 1985 to 1994, a sudden decline from 1995 to 1997 and yet again a rapid increase from 2000 to 2002 followed by a persistent decline.

The median age at diagnosis has increased throughout this follow-up; between 1973 and 1994 it was 1.2 years, and rose to 6.7 years from 2004 to 2009. The upward shift in age at diagnosis can partly be explained by the fact that the epidemic birth cohorts of 1985–1995 are getting older and are carrying along an excess risk. However we also notice a similar pattern in the post epidemic birth cohorts thus suggesting additional exposures that are probably related to modern life-styles and are yet to be identified. Increased awareness of CD symptoms that are typical in older children, as well as screening of siblings, could have contributed to the shift towards an older age at diagnosis, but the role of additional environmental factors should not be overlooked.

The variations in incidence among children below 2 years of age strongly suggest a role of environmental and/or lifestyle exposures that change over time. We have previously shown that infant feeding practices have played a crucial role in the onset and the end of a 10-year Swedish CD epidemic during the mid 1980s up until 1995 [6,7]. The continuing drop in incidence among children below 2 years of age began in 1995, coinciding with a one-third decrease in the average daily consumption of gluten-containing flour in children younger than 2 years of age, and introduction of the new national infant feeding recommendation that encouraged introduction of gluten in smaller amounts and preferably from 4 months of age with continued breastfeeding [6]. To date this recommendation remains in effect and most likely contributes to the continued CD decline in the youngest age group.

We previously showed a gap in the cumulative incidence between the epidemic and post-epidemic cohorts, but as both cohorts grow older, the gap continues to narrow. This is true regardless of whether cases were clinically detected or detected through screening [12,16]. In fact, during this follow-up, the cumulative incidence of the post-epidemic cohorts of 2000–2002 has surpassed that of the epidemic birth cohort (1985–1995) at certain ages. It is not yet clear as to why the 2000–2002 birth cohorts have a higher risk compared to other post-epidemic cohorts, since infant feeding guidelines have remained the same to date, thus stressing the role of other yet unidentified environmental risk factors.

Other environmental and lifestyle factors such as an increase in gluten consumption may explain the observed changes including the gradual increase seen over time among children 2–14.9 years, but can hardly explain the shorter and sudden changes that have been observed, especially in children below 2 years of age. The variations in the youngest children seemed to have an epidemic pattern both in period from 1985 to 1995 and the period from 2000 to 2002 and therefore are more likely to be associated with periodical changes such as episodes of infections [8,27,28]. Interestingly, during the Swedish CD epidemic, rod-shaped bacteria were often found in the small intestinal mucosa of children with CD, but not in controls, and rarely in biopsies from later periods [29]. Changes in the intestinal microbiota have also been associated with CD susceptibility and might play a role in explaining our observations [30-32].

Another crucial factor that needs to be examined is whether the observed CD increase is due to increasing immigration. Sweden, just like other European countries, has experienced a positive trend of net migration from abroad. Nevertheless, it is unlikely that the demonstrated CD increase is due to increasing immigration, because the majority of the immigrant population comes from low CD-prevalent countries. During the 1980s, immigration was mainly from Iran, Lebanon, Poland and Turkey. In the 1990s immigration was mainly from the former Yugoslavia, and in the 2000s from Iraq, Somalia, Afghanistan and Ethiopia [33]. Moreover, a recent study of worldwide immigration in Sweden showed non-European ethnicities to have a much lower CD susceptibility compared to the native population [34].

## Conclusion

In conclusion, this study demonstrates a significant rise in the incidence of childhood CD from 1973 to 2009. From following the child population of CD in Sweden for 36 years we have learned that CD incidence seems to be heavily influenced by changes in the environment and lifestyles. This implies that CD research should prioritize identification of new environmental agents that could contribute to new preventive strategies in addition to infant feeding, which is already implemented in both Europe and the US [35].

## Abbreviations

CD: Celiac disease.

## Competing interests

There are no competing interests to disclose.

## Authors’ contributions

AI, CO and OS were responsible for the original study design and data collection. NF and ML prepared data for analysis and jointly analyzed the data. NF wrote the first draft of the manuscript and all authors contributed in interpretation the study findings, consecutive revisions and all approval this submitted version of the manuscript.

## Pre-publication history

The pre-publication history for this paper can be accessed here:

http://www.biomedcentral.com/1471-230X/14/59/prepub
